# The Relationship Between Surprisal and Prosodic Prominence in Conversation Reflects Intelligibility‐Oriented Pressures

**DOI:** 10.1111/cogs.70134

**Published:** 2025-10-27

**Authors:** Thomas Hikaru Clark, Moshe Poliak, Tamar Regev, A. J. Haskins, Caroline Robertson, Edward Gibson

**Affiliations:** ^1^ Department of Brain and Cognitive Sciences Massachusetts Institute of Technology; ^2^ Department of Psychological and Brain Sciences Dartmouth College

**Keywords:** Surprisal, Prosodic prominence, Uniform information density, Audience design, Probabilistic reduction, Backchannels, Conversation, Large language models

## Abstract

Conversation is a dynamic, multimodal activity involving the exchange of complex streams of information like words, prosody, gesture, eye contact, and backchannels. Understanding how these different channels interact in naturalistic scenarios is essential for understanding the mechanisms governing human communication. Past studies suggested that the duration of words is tied to their predictability in context, but it remains unclear whether this relationship is speaker‐oriented (e.g., retrieval or production‐based) or due to listener‐oriented, intelligibility‐based pressures (i.e., emphasizing unpredictable words to ease comprehension). This study aims to examine the relationship between predictability and additional acoustic variables, to test how much intelligibility‐oriented principles impact conversation. We use the GPT‐2 large language model to assess the relationship between surprisal, a measure of unpredictability, and several variables known to play an important role in conversation—the prosodic features of duration, intensity, and pitch. We perform this analysis on the CANDOR corpus of naturalistic spoken video call conversation between strangers in English. In keeping with previous results using n‐gram predictability, we find that GPT‐2 surprisal predicts significantly higher values for duration. Moreover, surprisal also predicts maximum pitch and pitch range even when controlling for duration, with mixed evidence for an effect of surprisal on intensity. Additionally, we investigated listener backchannels (short interjections like “yeah” or “mhm”) and found that listener backchannels tended to be accompanied and followed by a spike in the surprisal of speakers' words. Finally, we demonstrate a divergence between the effect of context window size on the model fit of surprisal to maximum pitch and to other variables. The results provide additional support for intelligibility‐based accounts, which hold that language production is sensitive to a pressure for successful communication, not just speaker‐oriented pressures. Our data and analysis code are shared: https://osf.io/sqpn6/?view_only=e4d9e36c68b54863bc781e359463e1fe.

## 1 Introduction

Conversation involves the coordination of multimodal channels of information between two or more individuals in real time. As we share information with a conversation partner, we also dynamically modulate the rate of our speech, the pitch of our voice, and various nonverbal cues (e.g., eye‐contact, gestures, and facial expressions). What explains speakers' and listeners' modulation of these cues, and how (if at all) do these cues help conversational partners communicate successfully?

It is natural to think that speakers coordinate what they say and how they say it during conversation in order to facilitate robust communication with their conversation partner. However, whether such coordination reflects listener‐oriented or speaker‐oriented pressures is currently unclear (Arnold, 2008; Wagner & Watson, 2010). A notable case study is the predictability‐duration relationship: prior work has shown that words that are more predictable in their preceding linguistic context (e.g., “read the **book**” vs. “drop the **book**”) tend to be spoken with reduced duration (Bell et al., 2009; Jurafsky et al., 2001; Seyfarth, 2014). Similar relationships have been found for repeated words (Jacobs et al., 2015; Kahn & Arnold, 2015) and for syllables (Ibrahim et al., 2022). One interpretation of this finding is listener‐oriented: speakers might intentionally slow down and increase prominence for words that are less predictable in the context of the conversation for the benefit of their conversation partner, to smooth the flow of information and facilitate comprehension for the listener (Aylett & Turk, 2004, 2006; Frank & Jaeger, 2008; Jaeger, 2010; Levy & Jaeger, 2006; Pate & Goldwater, 2015). This account is also known as the intelligibility‐based account, since it emphasizes strategies speakers employ to make their intended message more intelligible for a listener (Galati & Brennan, 2010; Gahl et al., 2012; Jaeger & Buz, 2017). An alternative account for the predictability–duration relationship is speaker‐oriented: unpredictable words are simply more difficult to retrieve in the mind of the speaker, leading to slowed production (Bell et al., 2009; Gahl et al., 2012). In summary, both the listener‐oriented and speaker‐oriented accounts suggest that more predictable words should be reduced in duration and that more surprising words should be longer in duration, but for different reasons. Additionally, some combination or interaction of listener‐ and speaker‐oriented processes may be at play (Arnold et al., 2012; Arnold & Watson, 2015).

Recent studies lend support to the listener‐oriented account by considering additional dimensions of prosodic prominence beyond duration, such as pitch and intensity. Under a purely speaker‐oriented account, the predictability–duration relationship is explained by higher retrieval latencies for unpredictable words rather than intentional, listener‐oriented emphasis of unpredictable words; this account, therefore, does not explicitly predict a positive effect of unpredictability on pitch and intensity when controlling for duration. Under a listener‐oriented account, however, multiple dimensions of prosody may be used to render more important words more prominent. Previous work has linked reduced accessibility for words to a range of prosodic prominence measures, but it has been difficult to dissociate this association from intelligibility‐oriented pressures (Arnold, 2008; Wagner & Watson, 2010). At the same time, some work from the second language literature indicates that speakers may be more likely to mumble—that is, *reduce* prominence—for words which are difficult for them to say (Dörnyei and Scott, 1997). Thus, in the absence of clear mechanisms directly linking retrieval and production difficulties to increased pitch and intensity, we believe that observing a positive effect of surprisal on pitch and intensity (while controlling for duration) is more consistent with a listener‐oriented interpretation. Under this framework, two recent findings provide evidence for a listener‐oriented account. First, in a corpus study of English audiobooks, word *surprisal* (i.e., negative log probability) correlated with a composite measure of prosodic prominence that includes duration, pitch, and intensity (Wolf et al., 2023). This suggests that speakers coordinate predictability and multiple aspects of prosody while reading aloud, consistent with a listener‐oriented account, raising the question of whether such a relationship is also found during natural dyadic conversations. This is partially answered by a second recent study of dyadic conversations in Mandarin Chinese, which showed that the predictability of a word as well as its average informativity (its predictability across all contexts in which it appears) predicts pitch and intensity (Tang & Shaw, 2021). Yet, it remains unknown whether this predictability‐prosody relationship would extend beyond tonal languages, where exaggerating the pitch of unpredictable words could disambiguate minimal pairs differing only in tone. In sum, it is currently unclear whether the correlation between a word's predictability in context and the acoustic features of prosodic prominence are best accounted for by speaker‐ versus listener‐oriented pressures during natural, real‐world conversations. Further, whether such a predictability‐prosodic relationship is reflected in response cues from the listener is currently unknown.

Here, we sought to understand the speaker‐ versus listener‐oriented pressures that underlie how partners coordinate linguistic and nonlinguistic cues during conversation in a large corpus of dyadic conversations. We asked two main questions. First, we asked to what degree word predictability explains different dimensions of prosodic prominence during naturalistic conversation. We hypothesized that word predictability explains variance in prosodic prominence beyond just duration in natural English conversations. Second, we asked to what degree word predictability explains listener behavior, as indexed by backchannel signals (i.e., short interjections such as “yeah” or “mhm” that signal a listener's engagement), which are known to play an important role in conversation (Gravano & Hirschberg, 2009; Gravano et al., 2012; Jurafsky et al., 1997; Knudsen et al., 2020; Meyer, 2023; Nguyen et al., 2024; Tolins & Fox Tree, 2014; Ward & Tsukahara, 2000; Yngve, 1970). If the predictability‐prosody relationship exists in service of the listener, the listener might also coordinate backchannel signals to reflect this relationship (e.g., using backchannels in response to more surprising words spoken by the conversational partner). To test these hypotheses, we leverage the CANDOR corpus—a large, recent, audio‐video dataset of dyadic conversations between strangers in English (Reece et al., 2023), which is well‐suited for shedding light on the predictability–prosody relationship.

To foreshadow our results, we found that surprisal predicts prosodic prominence, including maximum pitch and pitch range even when controlling for duration, during natural, dyadic conversation (Appendix A, Tables A1, A2, A3, A4). Specifically, words that are more surprising in context tend to be expressed with increased prosodic prominence by the speaker. Prosodic prominence as indexed by maximum intensity, on the other hand, did not show a consistent effect from surprisal. Turning to backchannels, we found that backchannels were associated with elevated surprisal immediately before, during, and immediately after the use of the backchannel. This is consistent with speakers potentially responding to backchannels by introducing novel information, as has previously been suggested (Bergey and DeDeo, 2024). The slight increase in surprisal immediately before a backchannel suggests that listener backchannels may preferentially be used in response to surprising or informative material by the speaker, though this would imply a very low latency between a speaker's use of a surprising word and a listener's decision to backchannel.

In summary, our results suggest that speakers emphasize words when they are harder to predict, not just in the dimension of word duration, but in pitch as well. Given that this is difficult to explain with a solely speaker‐oriented account, we conclude that intelligibility‐oriented pressures play a role in this observed modulation of a speaker's prosody during conversation. At the same time, listener backchannels may also serve a communicative function by eliciting the introduction of novel information by the speaker.

## 2 Materials and methods

### 2.1 Data

**Fig. 1:**
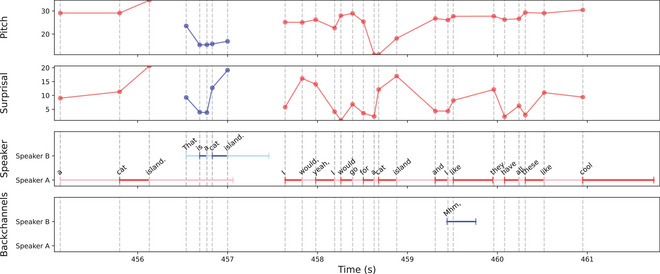
Example snippet of a conversation in Candor, with transcriptions and annotations of selected variables.

We employ the CANDOR dataset, which consists of video call conversations conducted in 2020 between pairs of strangers (Reece et al., 2023). The total dataset consists of approximately 1600 conversations, each lasting at least 25 min. Participants were English speakers (the CANDOR dataset does not specify whether they are native English speakers) living in the United States. They were not given any specific topic to discuss, and were simply instructed to “talk about whatever you like — just imagine you met someone at a social event and you're getting to know each other” (Reece et al., 2023). The dataset contains the raw audio and video recordings of the conversation (from both participants), automatically generated transcripts of the speech (using AWS Transcribe), and questionnaires about the conversation and the conversation partner that each participant filled out. Fig. 1 shows a visualization of the various streams of information present in the CANDOR dataset.

We divided the dataset into exploratory and confirmatory partitions of 50 conversations each. Our exploratory partition consisted of 50 conversations, which we used to validate our pipeline for feature‐extraction and conduct initial exploratory data visualization and analysis (97 unique participants; ages 19–63, median 33; 51 female, 41 male, 5 other/no answer). Our confirmatory partition consisted of 50 randomly sampled conversations, excluding conversations in the exploratory partition (98 unique participants; ages 19–65, median 33; 49 female, 46 male, 3 other/no answer). One participant was excluded due to not providing an age, which was a predictor in our analysis. In total, each of these partitions corresponded to approximately 21 h of audio conversation.

### 2.2 Computing surprisal

In previous studies, predictability has typically been computed based on either the previous word, that is, $p(w_{i}|w_{i-1})$, or the following word, that is, $p(w_{i}|w_{i+1})$ (Bell et al., 2009; Seyfarth, 2014; Tang & Shaw, 2021). Modern large language models (LLMs) now provide a way to approximate the predictability of words in context using neural networks trained on vast quantities of text, and with much larger context windows than the n‐gram models of prior work (Devlin et al., 2019; Radford et al., 2019). Large language models have been shown to capture human‐like syntactic generalizations (Hu et al., 2020) and to have internal representations that are correlated with human neural activity (Schrimpf et al., 2021). A language model takes in a linguistic string and assigns a probability to each unit within the string (typically words or sub‐word tokens). We compute surprisals for every word in a conversation using the GPT‐2 autoregressive (i.e., left‐to‐right) language model. Despite GPT‐2 being a relatively small model compared to the current state‐of‐the‐art LLMs, it has been shown to be a better predictor of human reading times than much larger models trained on much more data (Shain et al., 2024), which suggests that it occupies a “sweet spot” in the relationship between model performance and psychometric predictivity (Goodkind & Bicknell, 2018; Oh et al., 2022; Wilcox et al., 2023). We calculate surprisal on a word‐by‐word basis, as separated by whitespace; we use the surprisal correction of Pimentel and Meister (2024) to account for the bias in surprisal values introduced by sub‐word tokenization and leading whitespace (see also Oh and Schuler, 2024).

**Fig. B1:**
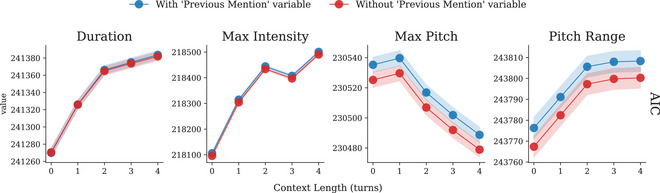
Comparison of Akaike Information Criterion (AIC) for different response variables, with and without “previous mention” variable. Results are shown for analysis on content words only. Lower AIC values indicate better model fit. Shaded bands denote a region of 5 units on either side of a point; a difference in AIC of 10 is considered “substantial” (Burnham and Anderson, 2004). We observe that the presence of a “previous mention” predictor does not yield lower AIC scores than models fit without this predictor, and in fact leads to numerically higher AIC values across all predictors and context lengths, though the differences are not large enough to be considered substantial. While past studies of probabilistic reduction included previous mention as a control predictor due to using very short‐context n‐gram language models, based on these results, we argue that previous mention may be subsumed by surprisal when the language model has sufficient context.

In contrast to the small context window of n‐gram models, the GPT‐2 model we use has a maximum context window of 1024 tokens (Radford et al., 2019). We consider a range of context lengths in terms of conversational turns, from 0 turns (indicating that the model only has access to the current turn), to 4 turns (indicating that the previous 4 turns of conversational context are provided as context to the language model). In the case of very long previous turns, context was truncated at the maximum context window size for GPT‐2 of 1024 tokens. However, 91.6% of turns had 50 words or fewer, so, in practice, this was not a concern. While the concept of a “turn” has no universally accepted definition, we employ the Backbiter strategy for delineating turns, as defined in the CANDOR dataset (Reece et al., 2023); this involves defining a turn boundary whenever there is a change of speaker while ignoring backchannel utterances. Past studies of probabilistic reduction typically used only bigram models to quantify predictability, meaning that the probability of a word in context was only calculated conditioned on the immediately previous (or immediately following) word. As a result, past studies included additional predictors such as previous mention of a word in the conversation (Seyfarth, 2014; Tang & Shaw, 2021), which were included to reflect the fact that longer‐range context can influence word predictability (e.g., words which have been previously mentioned are more likely to be mentioned again). We take the GPT‐2 surprisal predictor as subsuming other predictability‐related variables computed over its context window, and by testing multiple context window sizes, we can estimate the influence of long‐ and short‐range context on the predictability effect. In Appendix B, we compare regression models fit with and without an explicit previous mention variable, finding that including previous mention does not improve model fit (Fig. B1).

#### 2.2.1 Handling of punctuation

The automatically generated CANDOR transcripts contain punctuation such as commas and periods. We compute surprisal on the transcripts exactly as they are, without removing punctuation. Applying the method of Pimentel and Meister (2024) yields surprisal values for each whitespace‐separated word, including any punctuation that is included without a space. However, this means that words with punctuation will tend to have higher surprisal than words without, all else being equal, despite the fact that punctuation is an artifact of the automated transcription, not something observable directly in the speech stream. As a result, we exclude any words that are connected to punctuation from our regression analysis.

### 2.3 Prosodic features

**Fig. C1:**
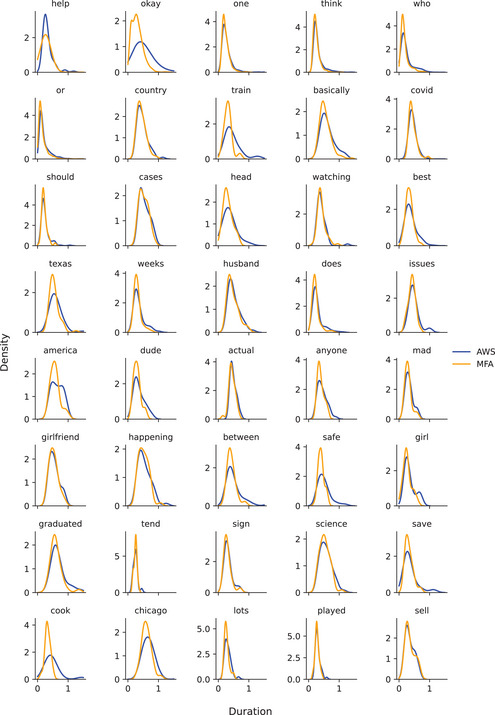
Density plots of word duration for 40 words randomly sampled from the 1000 most frequent words in our analysis's subset of CANDOR. We observe that the duration values derived from AWS Transcribe timestamps tend to have slightly heavier tails for longer duration values, compared to the values derived from the Montreal Forced Aligner.

We quantify the following prosodic features for each word in a conversation: duration, maximum intensity, maximum pitch, and pitch range. We use the Montreal Forced Aligner (MFA) (McAuliffe et al., 2017) to extract start and end timestamps for each word in the dataset (alignment is performed within turns using each turn's automated transcription and the corresponding audio). In Appendix C, we compare the extracted durations for words according to MFA and the timestamps from AWS Transcribe provided in CANDOR (Fig. C1). We use the Parselmouth library (Jadoul et al., 2018), which is built on top of the Praat software (Boersma and van Heuven, 2001), to extract intensity and pitch features for each word based on the start and end times for the word and the raw audio, considering only the audio channel corresponding to the current turn's speaker. Maximum intensity is measured in decibels (dB SPL), a logarithmic measure of a sound's energy above a minimum perceptible threshold. We measure maximum pitch in semitones above a reference value of 50 Hz. This ensures that perceptually similar anomalies from different baselines (e.g., an increase in pitch by an octave) would be given the same rating. We measure pitch range as the change in semitones between the maximum and minimum pitch detected within the span of a word.

### 2.4 Backchannels

We employ the Backbiter transcription provided in CANDOR, in which short utterances containing backchannel words such as “yeah” and “mhm” are not marked as separate turns, but are placed in a separate backchannel column in the transcript. This contrasts with a naive model of turn‐taking in which every interjection is considered a new turn, which would lead to highly fragmented conversations. In the original Backbiter transcript, each turn is coded with a single backchannel onset timestamp (defined as the start of the first‐occurring backchannel in the turn) and a single backchannel offset timestamp (defined as the end of the last‐occurring backchannel in the turn); this coding, therefore, does not provide onset and offset timestamps for individual backchannels in cases where multiple backchannels exist in a turn. We aligned this backchannel information with the underlying raw transcription file to get precise start and end timestamps for each separate backchannel utterance, allowing us to conduct an analysis in which we examine the surprisal of speakers' words when time‐locked to the start of each listener backchannel. We only include words with backchannel overlap that have five words on either side within the same speaker's turn (in order to be able to visualize the time‐course of surprisal before and after a backchannel). We show two conditions, one where we exclude words that are transcribed with following punctuation (e.g., commas or periods), and one where we include such words. We note that the presence of punctuation will tend to increase the surprisal of a word under a language model. We predicted that we might see a spike in surprisal preceding backchannels, if listeners use backchannels to acknowledge a surprising or informative word.

### 2.5 Mixed‐effects regression

A word can appear in many different contexts, where it will have different levels of predictability, but crucially, constant values for any word‐intrinsic features such as word frequency or number of syllables. A strong test of whether surprisal predicts a given word's prosodic prominence is whether variance in prominence among different instances of the same word can be explained by surprisal.

To address this question, we perform a linear mixed‐effects regression to predict prosodic features for each word using the lme4 ^1^ package in R, using a formula of the form:

duration ∼ surprisal + frequency + numberOfSyllables + speakerAge + speakerSex + durationBaseline + speechRate + preWordPause + previousMention + hasPunctuation + (surprisal | word) + (surprisal | speaker) + (1 | wordsFromStart) + (1 | wordsFromEnd)

or

{maximumPitch, pitchRange, maximumIntensity} ∼ surprisal + frequency + speakerAge + speakerSex + acousticBaseline + duration + speechRate + preWordPause + previousMention + hasPunctuation + (surprisal | word) + (surprisal | speaker) + (1 | wordsFromStart) + (1 | wordsFromEnd)

For a given prosodic response variable, that is, duration, maximum pitch, pitch range, or maximum intensity, we fit a regression with fixed effects of surprisal and several control predictors. We include random intercepts for each word and for each speaker, and by‐speaker and by‐word random slopes for the surprisal variable. This random‐effects structure helps to account for pitch and intensity variation across speakers (due to either their unique vocal features or their computer microphone), as well as acoustic variation across words due to differences between speech sounds.

For all response variables, log word frequency (per million corpus words, calculated based on the SubtlexUS movie subtitles dataset (Brysbaert and New, 2009)) was included as a control predictor (all tokens of the same type have the same value, and the alignment of words in CANDOR to words in SubtlexUS was case‐insensitive). The duration of pause preceding a word was included as a control predictor. Speaker speech rate (each speaker's average number of syllables spoken per second, across all utterances from the speaker) was included as a control predictor. Acoustic baselines were computed using a leave‐one‐out average: for each word token and for each of the acoustic variables of duration, max intensity, max pitch, and pitch range, the average value of the acoustic variable across all other tokens of the same type was computed and included as a baseline predictor. The position of a word in a turn relative to turn start and turn ending were added as random intercepts; this is intended to capture the effect of proximity to turn boundaries on the prosody of a word, which may be nonlinear in the distance from a turn boundary. Positions 1 through 9 (from either a turn start or turn ending) are the nonreference levels, while 10+ (10 or more words either from the turn start or turn ending) is the reference level. When predicting duration, the number of syllables in a word, as defined by the CMU dictionary in NLTK (Bird et al., 2009), was included as a control predictor. If the word was not present in the CMU dictionary (e.g., for names, locations, or nonwords), we use the number of possible hyphen insertion positions from the Pyphen package (Kozea, 2023) as a fallback.

Words which occurred less than five times in the dataset were dropped from the analysis to avoid a long tail of low‐frequency words which could cause convergence issues with fitting per‐word slopes and intercepts. Words which did not occur in the SubtlexUS word frequency corpus were also dropped. This resulted in a total of 216,392 observations (words). All continuous variables were centered and scaled prior to model fitting; thus, for our key predictor of surprisal, coefficients can be interpreted as the effect size (in standard deviations of the response variable) of a one‐standard‐deviation increase in surprisal.

To address the concern that the relationships under study may be driven largely by high‐frequency function words, we also repeated the analysis on only the content words in the dataset (97,087 observations). Content words were defined by excluding the set of English stopwords in the Python Natural Language Toolkit (NLTK) library (Bird et al., 2009) and the following filler and backchannel words: *oh*, *uh*, *um*, *yeah*, and *like*. All control variables and model formulas were otherwise identical.

## 3 Results

### 3.1 Higher surprisal predicts increased prosodic prominence beyond duration

**Fig. 2:**
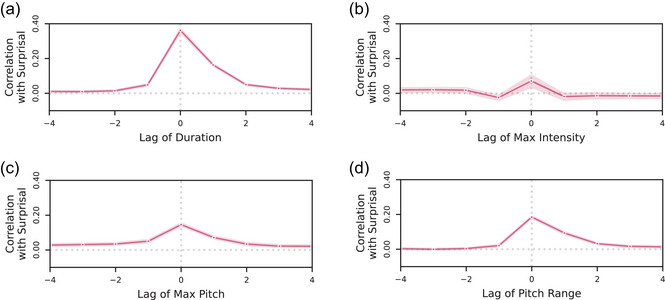
Cross‐correlation between surprisal and prosodic response variables. These are relationships between the raw data; regression model outputs are shown in Tables A1, A2, A3, A4. Error bands denote 95% confidence intervals computed across conversations. Concretely, a lag of k indicates that the response variable stream is shifted by k units relative to the surprisal stream such that the surprisal of the $i^{th}$ word is aligned with the response variable of the ${\left(i-k\right)}^{th}$ word. There is a noticeable spike in correlation between a word's surprisal and each of the prominence measures, while the features vary in the degree of spillover of this correlation onto neighboring words.

To visualize the relationship between linguistic surprisal and prosodic prominence, we first compute the correlation between a word's surprisal and the following features of prosodic prominence: duration, maximum intensity, maximum pitch, and pitch range (all prosodic features were computed at the single‐word level). Correlations between surprisal and each prosodic feature are computed with a sliding offset value to visualize the time‐course of this correlation (i.e., whether a word's surprisal correlates not just with the same word's prosody, but with past or future words' prosody). Correlations are aggregated across conversations within the dataset, yielding an overall mean and 95% confidence intervals. Fig. 2 shows the resulting cross‐correlation plots, which show a positive correlation between surprisal and each of the prosodic variables. Furthermore, the correlations quickly fall off to near zero when the surprisal values and prosody values are not aligned, indicating that the surprisal of a specific word is much more tightly coupled to the prosody of that specific word, as opposed to generally related to other words in the same vicinity.

To further investigate these correlations, we fit linear mixed‐effects regression models to predict the prosodic feature of interest (i.e., duration, intensity, pitch) using surprisal while controlling for additional variables (full model formulas and additional details are provided in *Materials and methods: Mixed‐effects regression*). For the prosodic variables of duration, maximum pitch, pitch range, and maximum intensity, the effect of surprisal was positive (Duration: β=0.023, SE=0.004, p≪.001; Max Pitch: β=0.041, SE=0.007, p≪.001; Pitch Range: β=0.041, SE=0.004, p≪.001; Max Intensity: β=0.019, SE=0.006, p=.002; reported coefficients are when using surprisal from language models with the maximum context length of 4 turns). For the prosodic variable of max intensity, we note that the results were more mixed than for the other prosodic variables —for regressions performed using different context lengths, the effect had a mixed pattern of significance at the p=.05 level. Importantly, we detect an effect of surprisal on maximum pitch and pitch range, even when controlling for duration, which was a positive and significant predictor of each of these variables (Max Pitch: β=0.106, SE=0.003, p≪.001; Pitch Range: β=0.217, SE=0.003, p≪.001; Max Intensity: β=0.109, SE=0.003, p≪.001). Full regression model outputs are provided in the Appendix, Tables A1, A2, A3, A4.

Back‐converting the coefficients into the original units, we arrive at the following effects of surprisal on prosodic variables: effect of surprisal on duration: 2 ms/bit, effect of surprisal on max pitch: 0.11 semitones/bit, effect of surprisal on pitch range: 0.11 semitones/bit, effect of surprisal on max intensity: 0.02 dB/bit. The standard deviation of the surprisal variable is approximately 3.1 bits; thus, a surprisal difference of 3 standard deviations between two occurrences of the same word could be expected to result in approximately 16 ms in increased duration, 1 semitone in increased pitch, 1 semitone in increased pitch range, and 0.2 dB in increased intensity. Full regression model outputs considering content words only are also provided in the Appendix, Tables A1, A2, A3, A4. Limiting the analysis to only content words produced qualitatively similar results.

**Fig. 3:**
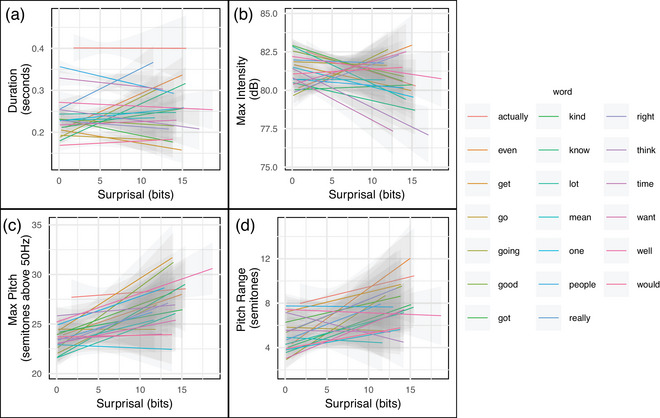
Relationship between surprisal and four response variables for 20 most frequent content words in the dataset. Content words were defined by excluding the set of English stopwords in the Python Natural Language Toolkit (NLTK) library (Bird et al., 2009) and the following filler and backchannel words: *oh*, *uh*, *um*, *yeah*, and *like*. Within each panel, each line corresponds to a unique wordform, which may appear in many different contexts. Crucially, even when examining within individual words, a positive association between surprisal and prosodic prominence is evident for the dimensions of duration, max pitch, and pitch range; for max intensity, the pattern is mixed.

Fig. 3 shows the relationship between surprisal and prosodic features for individual words (the 20 most common content words in the dataset are shown). The same word, when used in different contexts, differs in both its surprisal and its average prosodic prominence, and the majority of individual words exhibit a positive correlation between these two variables. The examination of content words here suggests that the relationship between surprisal and prosodic prominence is not driven purely by short, reduced, and predictable function words, but rather reflects a context‐sensitive modulation of the prosodic prominence of words. Qualitatively, we note that the relationship among the selected words appears less consistent for the max intensity variable. This is consistent with the mixed results found in the mixed‐effects regressions.

### 3.2 Relationship between word surprisal and listener backchannels

**Fig. 4:**
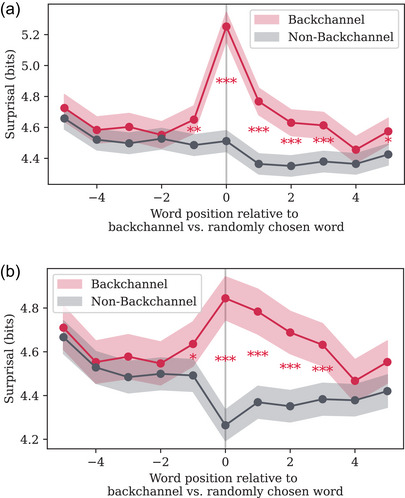
Speaker surprisal time‐locked to each backchannel occurrence with at least five preceding and five following words in a conversational turn. Position 0 denotes the word which overlaps with the start of the backchannel (the “critical word”). For comparison, surprisal time‐locked to randomly chosen non‐backchannel words (words uniformly sampled with probability 0.05, excluding words overlapping with backchannels) is shown. No previous turns of context were used for the calculation of surprisal (note that within our dataset, where surprisal was computed using anywhere from 0 to 4 previous turns of context, surprisal values were very highly correlated for all pairs of context lengths, with a minimum value of 0.914 and a maximum value of 0.99). In 4a, no restrictions are placed on the critical word. In 4b, words which contain punctuation are excluded, which results in lower surprisal at the critical word. Stars indicate the significance level of a *t*‐test comparing the mean surprisal at each word position in the Backchannel condition against the Non‐Backchannel condition, with a false discovery rate correction. In the presence of backchannels, there is a spike in surprisal, with a significant difference between the Backchannel and Non‐Backchannel conditions starting one word before the critical word, spiking sharply, then decreasing.

In addition to the relationship between surprisal and prosodic prominence, a secondary research question in this study pertains to listener behavior: are *listeners* more likely to produce backchannel utterances in response to surprising words? Fig. 4 shows time‐locked surprisal values as a function of position relative to a backchannel. Values in the Backchannel condition are compared to values in the Non‐Backchannel condition using a *t*‐test with false discovery rate correction assuming positive correlation between time positions and a threshold of p=.05. We observe a significant increase in surprisal in the Backchannel condition relative to the Non‐Backchannel condition, immediately before, during, and after the backchannel, with surprisal values then returning to the Non‐Backchannel baseline level within a few words. This temporary spike in surprisal could have several possible interpretations, which we address in the Discussion, but it suggests that speakers tend to respond to listener backchannels by producing words which are not as predictable as would otherwise be expected.This result is partially consistent with one existing work (Bergey and DeDeo, 2024), who similarly found that backchannels tended to trigger the introduction of novel material by the speaker, but also found that surprisal tended to decrease in the lead‐up to a backchannel.

### 3.3 Role of language model context length in predicting prominence

**Fig. 5:**
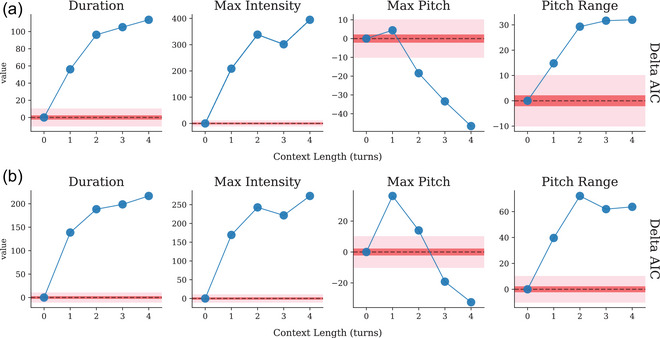
Delta AIC scores comparing model fit when using surprisal from language models with access to 1–4 additional conversational turns of context in comparison to the baseline model (current turn only). Lower AIC values indicate a better fit to the data. A difference in AIC of greater than 10 in either direction (pink region) is considered substantial (Burnham and Anderson, 2004). For duration, intensity, and pitch range, surprisal computed using additional turns of context predicts prominence less well, while for max pitch, surprisal computed with additional turns of context predicts prominence better.

The predictability of a word is sensitive to how much context is provided, and what the context is; the final word of the sentence *“She fills the bucket with fish.”* is less surprising when the preceding sentence is *“The zookeeper is preparing to feed the seals.”*, compared to when no previous context is provided. Thus, the context window size (here measured in number of preceding conversational turns) can affect the surprisal of a word (e.g., by providing additional context that changes the language model's estimated probability distribution over the given word). While it has been established that predictability influences a speaker's prosody in conversation, it remains unclear whether the human sensitivity to predictability is best modeled by using short‐range or long‐range context, though existing work suggests that there are meaningful differences on the scale of a few words (Regev et al., 2025). Additionally, other work has suggested that across multiple languages, surprisal from language models with *shorter* context windows are better predictors of human reading times than models with longer context windows, when controlling for architecture (Kuribayashi et al., 2022, 2024). We investigated this question by comparing the model fit of linear mixed‐effects regression models differing only in the amount of context provided to the language model when computing surprisal (using a range from 0 to 4 previous conversational turns). Models were compared using the Akaike Information Criterion (AIC), where lower values indicate a better fit of the model to the data (Fig. 5). We note that each model was fit on the same number of observations with the same number of model parameters, with all predictors besides surprisal being held constant. Our analysis revealed a mixed pattern of results. For all variables except max pitch, the surprisal values computed using longer‐range context were *worse* predictors of prominence than surprisal computed using only the current turn. For max pitch, the opposite pattern held: AIC reached a peak at 1 turn of context, then decreased when additional turns of context were added to the computation of surprisal.

## 4 Discussion

### 4.1 General discussion

In this paper, we investigated how word predictability relates to different dimensions of prosodic prominence and listener behavior during naturalistic conversation, as a way of assessing the influence of listener‐oriented, communicative pressures on conversation. In a corpus of open‐ended, dyadic conversation, we found that the surprisal (negative log probability) of a word in context (as measured by the GPT‐2 large language model) is positively correlated with the word's duration, pitch, and intensity—three measures of prosodic prominence. Language model surprisal was a significant predictor of max pitch and pitch range even when controlling for duration. Interestingly, the estimates we found for the effect of surprisal on duration are slightly lower but of the same order of magnitude to the reported effect of surprisal on reading times from the psycholinguistics literature (Smith & Levy, 2013; Wilcox et al., 2023). This suggests that the magnitude of the slowdown incurred per unit of surprisal is roughly on par across comprehension and production. Additionally, we found that language model surprisal is also coupled with listener backchannels (brief interjections like “yeah” or “mhm”)—the surprisal of words from a speaker tends to increase immediately before, during, and after a backchannel. Our findings suggest that multiple dimensions of speaker behavior are sensitive to the predictability of words in the conversation; we now discuss the implications of these findings in the context of the literature.

Our findings tie into a rich literature on the role of information‐theoretic principles—and in particular, the pressure for robust and efficient communication—in shaping human language. According to the Uniform Information Density hypothesis (UID) (Jaeger, 2010; Levy & Jaeger, 2006) and related theories such as the Smooth Signal Redundancy hypothesis (SSR) (Aylett & Turk, 2004, 2006), language producers tend to spread information smoothly and uniformly in a given linguistic signal, adopting various strategies to avoid large spikes or troughs in surprisal. For example, speakers have a greater tendency to insert optional linguistic units, such as the word “that” in the sentence “I liked the movie [that] you recommended,” when upcoming material is unpredictable in context; this has the effect of spreading out new information, which avoids excessive cognitive load on the comprehender, and makes the language signal more robust to noise, thereby increasing intelligibility and maximizing the chances of communicative success (Jaeger, 2010; Levy & Jaeger, 2006). Related theories such as SSR have proposed that a pressure for information uniformity affects not only what words people choose to say, but *how* they choose to say them. For example, prior work on the predictability–duration relationship has shown that highly predictable words tend to be reduced in spoken production, which suggests that speakers modulate their speech rate to keep information density relatively uniform (Aylett & Turk, 2004, 2006). Recent works have also argued for an influence of information uniformity on other, diverse linguistic phenomena such as incremental reading times (Meister et al., 2021) and word order rules (Clark et al., 2023).

At the same time, the UID hypothesis has come under scrutiny based on some negative results and a lack of clarity on how to operationalize “uniformity” (see Juzek (2024) for a critical review). Even in cases like the predictability–duration relationship in spoken production, where UID offers a plausible explanation, the phenomenon can be equally well explained by an alternative, speaker‐oriented account (which holds that unpredictable words are simply slower to retrieve and produce for a speaker) as by the listener‐oriented UID account (which argues that this correlation serves an intelligibility‐oriented function). These two explanations are not mutually exclusive, although some previous work on phonetic reduction has argued that speaker‐oriented pressures dominate listener‐oriented ones in words with high phonological neighborhood density (Gahl et al., 2012). Indeed, there are also aspects of language which appear to go against the principle of UID, such as jokes and creative metaphors, which typically correspond to local spikes in surprisal (Bunescu and Uduehi, 2022); UID is thus perhaps best interpreted as a general pressure on language, but one which is by no means absolute (Tsipidi et al., 2024).

In this work, we show that prosodic prominence is influenced by predictability even when accounting for the known effect of predictability on duration. This weighs against an exclusively speaker‐oriented account, as there is not a clear link between a word's difficulty in retrieval and it being produced with a higher pitch or louder volume, as there is for duration. We note that our results are still entirely consistent with the existence of speaker‐oriented pressures on production; we simply argue that the evidence also supports a theory of communication in which speakers modulate their prosody, especially their pitch, to selectively emphasize words which may be harder for a listener to predict, thereby facilitating comprehension (Aylett & Turk, 2004). In this sense, our results align with the general intelligibility‐oriented pressures proposed in the UID literature, without committing to any strict notion of uniformity per se as an absolute constraint. The fact that there are measurable relationships between how much information a word conveys in context, and its prosodic prominence, also offers a possible explanation for the observed redundancy between the prosody of spoken language and its purely textual representations (Wolf et al., 2023), and also generates novel testable hypotheses– ‐ that prosody may sound atypical or strange when less surprising words are pronounced more prominently than more surprising ones.

We now turn to the relationship between language model context length and the model fit of the surprisal–prominence relationship, in particular, the improvement in model fit when computing surprisal using additional turns of context for the max pitch variable but not for other variables, such as duration. One interpretation of this result is that the relationship between surprisal and duration can largely be explained using local context only. This is consistent with findings from the literature which established a relationship between predictability and duration using context windows of only a single word (i.e., bigram models) (Seyfarth, 2014; Tang & Shaw, 2021). The inclusion of longer‐range context appears to affect surprisal values in a way that makes them fit *less* well to word duration, intensity, and pitch range. It is possible that language models with access to long‐range context may *underestimate* the surprisal of a word as experienced by a human speaker, consistent with work showing that some language models can be “too good” at next‐word prediction for a match to human behavior, leading to worse fits to human reading times (Shain et al., 2024). However, this account is complicated by the fact that inclusion of longer‐range context improves the prediction of maximum pitch (but not pitch range). One possible explanation comes from work on linguistic constructions (Bybee and Brown, 2024), which has argued that the relationship between word duration and predictability is not necessarily a conscious adaptation on the part of speakers to in‐context predictability, but an effect of high‐frequency constructions: words are articulated in a reduced way when part of these high‐frequency constructions, but this effect is captured by short‐range contextual predictability rather than discourse‐level predictability. It is possible that this construction‐oriented account explains the model‐fit pattern for duration, intensity, and pitch range, while a distinct, intelligibility‐oriented pressure explains the pattern for max pitch. Indeed, from an intelligibility‐oriented perspective, it is reasonable to think that long‐range context would influence predictability, and that the words worth emphasizing are ones that are difficult to predict even with extended amounts of preceding context. This potentially points to two separate phenomena with differing explanations: on the one hand, probabilistic reduction of words' duration, intensity, and pitch range based on largely local relationships between words, and on the other hand, probabilistic emphasis using higher maximum pitch for words that are less predictable.

Given these results, an open question is whether speakers have any way of knowing which words a listener may find surprising, and what signals from a listener may exist to indicate their level of surprise. Our investigation of listener backchannels attempts to address this question. Far from passively waiting for their interlocutor's turn to end, it is well‐established that a listener produces backchannel utterances, which do not interrupt the flow of the speaker but provide a form of feedback from listener to speaker. These backchannels have been linked to prosody, narrative development, and other functions within conversation (Gravano et al., 2012; Jurafsky et al., 1997; Knudsen et al., 2020; Liu et al., 2022; Meyer, 2023; Nguyen et al., 2024; Tolins & Fox Tree, 2014; Ward & Tsukahara, 2000; Yngve, 1970). One study has considered the relationship between surprisal and backchannels, finding that backchannels are more likely to occur following dips in surprisal values, while surprisal tends to spike again immediately following a backchannel (Bergey and DeDeo, 2024). This suggested an impressive ability on the part of listeners to keep track of the information content of words. Our results indicate that surprisal tends to increase immediately before, during, and after a listener backchannel. This suggests that speakers may react to listener backchannels by introducing novel information or starting a new clause or idea, which would tend to increase surprisal temporarily. Our results differ from those of Bergey and DeDeo (2024) in finding a small but significant rise in surprisal immediately before words that overlap with backchannels (relative to randomly sampled non‐backchannel words), as opposed to a decrease in surprisal; we note that Bergey and DeDeo (2024) used a different language model and a different strategy for including conversational context in the computation of surprisal. Another explanation for these results is that listeners are attempting to wait for natural breaks in a speaker's turn in order to produce backchannels, but that these breaks are naturally followed by the introduction of new material by the speaker; however, our results show this pattern even when excluding backchannels that occur during words with end‐of‐sentence or end‐of‐clause punctuation (which tend to co‐occur with speakers' pauses).

### 4.2 Limitations

We now turn to some limitations of our work. First, these results and their interpretation depend on the assumption that a language model can assign probabilities to words in context in a way that aligns with human predictions. We employ the GPT‐2 language model, which has previously been shown to correlate strongly with human measures of processing difficulty, even more so than surprisal values from much larger language models such as GPT‐3 (Oh & Schuler, 2023; Shain et al., 2024; Wilcox et al., 2023). However, all language models are sensitive to the distribution of language within their training data. The conversational nature of the CANDOR dataset may, therefore, be out of distribution for the GPT‐2 model. We also note that language models may overestimate the surprisal of terms and concepts that become popularized or salient after the models were trained; for example, topics related to the COVID‐19 pandemic were attested in CANDOR but may be out‐of‐distribution for GPT‐2 (though we note that GPT‐2's training data contain a broad range of internet text). In this work, we have decided against training or fine‐tuning a language model specifically on conversational data for two reasons: first, evidence does not support the idea that language models with lower perplexities are necessarily a better match to human behavior, and second, training a custom model introduces considerable degrees of experimenter freedom (e.g., training dataset and hyperparameters) over using an off‐the‐shelf, widely used model with an established link to psycholinguistic features like reading time. Future work is needed to test whether our results generalize to different language models and conversation corpora, but our findings of significant fixed effects when including word‐ and participant‐level random effects suggest that the results were not driven by a small number of individual speakers or words.

**Fig. D1:**
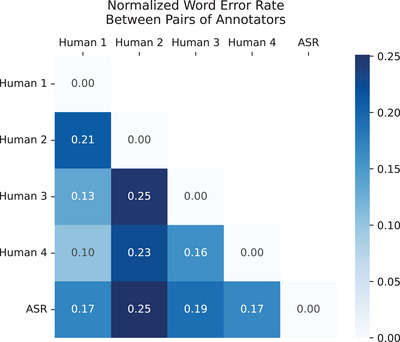
Pairwise evaluation of mean Word Error Rate (WER) for human annotators and CANDOR automated speech recognition (ASR) transcriptions. WER is normalized to values between 0 and 1, and is computed to be symmetric by averaging the two asymmetric WER values for each pair of transcripts. We observe that the CANDOR ASR transcription is generally on par with interhuman agreement—its average WER to the remaining transcripts is lower than that of the worst‐performing human annotator.

**Fig. D2:**
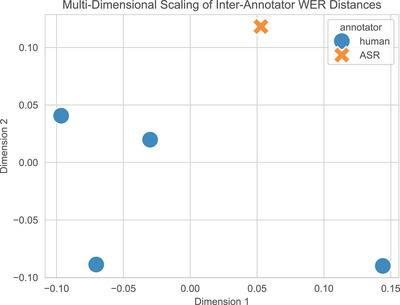
Multi‐dimensional scaling (MDS) visualization of WER distance between annotators. MDS attempts to show each annotator (four humans and one ASR) in two‐dimensional space, such that relative pairwise distances according to the WER distance matrix in Fig. D1 are preserved.

Second, there are limitations related to the data quality of the CANDOR corpus, which involved online recruitment and participation. Due to the nature of the dataset, acoustic features from conversations were recorded “in the wild” with participants' own devices, thus reducing audio quality relative to data collected in a laboratory environment (Sanker et al., 2021), such as the Buckeye corpus (Fosler‐Lussier et al., 2007). While we have accounted for speaker‐level variation in prosodic prominence using by‐speaker random intercepts, the dataset is still less than ideal, especially for evaluating the variable of intensity, which may vary with a speaker's distance from their microphone or other artifacts of recording. Additionally, while recordings in CANDOR were manually reviewed to filter out those with unusable audio quality, the CANDOR transcripts were automatically generated using the AWS Transcribe service rather than human‐annotated, possibly increasing the number of expected transcription errors (Stolcke and Droppo, 2017). However, we conducted a post‐hoc evaluation of a small subset of the CANDOR transcripts relative to four human annotators, and found that the mean word error rate of the automatic transcriptions was on par with the mean interhuman word error rate (Appendix D, Fig. D1 and D2). While transcription errors increase noise, we argue that they do not cause systematic bias in the direction of our observed effects. The most concerning scenario would be if words which are more prominent are more likely to be incorrectly transcribed, making them more likely to be assigned higher surprisal values (by virtue of being the “wrong word” for the given context); this could create a spurious correlation between prominence and surprisal. However, more prominent words are intuitively *less* likely to be incorrectly transcribed, as they have a louder and longer acoustic signal; we thus conclude that results are unlikely to be driven by a spurious correlation caused by transcription errors. Another issue with automatic transcriptions is that the CANDOR transcripts lack a manually coded variable for disfluencies in speech, found in some previous work (Bell et al., 2009; Seyfarth, 2014). One potential concern would be that disfluencies may be systematically more likely to be followed by words which are both high in surprisal and prosodically prominent, contributed to the observed surprisal–prosody relationship. While we acknowledge the limitation of lacking manually coded disfluencies, we argue that this pattern would not invalidate the main claim of this paper; in contrast, this would simply be one special case of an intelligibility‐oriented pressure to increase prosodic prominence at moments when listeners are most likely to need it.

An additional limitation is our study's exclusive focus on English, which limits its generalizability. We note that our results are generally consistent with findings from an analysis of Mandarin Chinese speech (Tang & Shaw, 2021), but future work can broaden the languages under study to see if similar patterns hold in languages with significantly different properties from English, such as pitch‐accent languages. Despite the limitations of the CANDOR dataset, its online recruitment conveys several advantages, such as a greater number of participants, allowing participants to converse in the comfort of their homes, and allowing participants to see each other, unlike telephone‐based conversational datasets like CALLHOME (Canavan et al., 1997) or Switchboard (Godfrey et al., 1992).

### 4.3 Conclusion

To conclude, this paper has investigated the relationship between predictability and prosodic prominence, finding that prosodic prominence is influenced by contextual predictability. These results lend support to intelligibility‐oriented accounts of human communication in which speakers modulate their prosody to emphasize informative words. Future work can consider additional signals in conversation, such as eye contact and gestures, which may also play a role in facilitating robust and successful communication. Additionally, it remains unknown what accounts for interparticipant variability in the strength of the relationship between prosody and predictability; future work can address the development of these patterns during language acquisition as well as their presence in clinical populations with differences in communication, for example, in autism spectrum disorder.

## Note

1 We considered the relative advantages and disadvantages of frequentist statistical modeling in lme4, versus Bayesian regression in brms. Advantages of the Bayesian approach include the ability to specify priors and brms returning full posterior distributions over model parameters, at the cost of greatly increased runtime. In this case, the number of observations is large ($∼10^{5}$), which means that the data likelihood tends to dominate over a Bayesian prior (e.g., Gelman et al., 2013), reducing the benefit of using brms relative to its increased runtime. Here, we use lme4 simply to produce estimates of main effect coefficients; however, in situations where capturing the uncertainty in the posterior distribution of model parameters is of particular interest, on a reasonably‐sized dataset, readers might consider using a Bayesian approach instead.

## Appendix A Regression results

**Table A1:** Regression results for predicting Max Intensity

Context Len.	Subset	Predictor	Estimate	Std. Error	Pr(>|t|)	Sig.
0	Content Words	(Intercept)	1.664	0.125	0.000	***
0	Content Words	surprisal	0.010	0.007	0.142	
0	Content Words	Word Frequency	−0.367	0.067	0.000	***
0	Content Words	Previous Mention	−0.001	0.007	0.876	
0	Content Words	age	0.009	0.011	0.393	
0	Content Words	Sex=Male	0.011	0.023	0.619	
0	Content Words	Sex=Other	−0.006	0.081	0.942	
0	Content Words	Max Intensity Baseline	−2.256	0.025	0.000	***
0	Content Words	duration	0.069	0.002	0.000	***
0	Content Words	Speech Rate	0.019	0.011	0.078	
0	Content Words	Pre‐Word Pause	−0.033	0.003	0.000	***
0	All Words	(Intercept)	0.101	0.051	0.061	
0	All Words	surprisal	0.013	0.007	0.063	
0	All Words	Word Frequency	0.035	0.005	0.000	***
0	All Words	Previous Mention	−0.017	0.006	0.007	**
0	All Words	age	−0.003	0.012	0.772	
0	All Words	Sex=Male	−0.016	0.024	0.524	
0	All Words	Sex=Other	−0.051	0.086	0.557	
0	All Words	Max Intensity Baseline	0.238	0.004	0.000	***
0	All Words	duration	0.109	0.002	0.000	***
0	All Words	Speech Rate	0.009	0.012	0.452	
0	All Words	Pre‐Word Pause	−0.034	0.003	0.000	***
4	Content Words	(Intercept)	1.662	0.126	0.000	***
4	Content Words	surprisal	0.014	0.006	0.011	*
4	Content Words	Word Frequency	−0.370	0.067	0.000	***
4	Content Words	Previous Mention	0.002	0.007	0.789	
4	Content Words	age	0.010	0.011	0.359	
4	Content Words	Sex=Male	0.002	0.022	0.938	
4	Content Words	Sex=Other	0.000	0.079	0.998	
4	Content Words	Max Intensity Baseline	−2.251	0.026	0.000	***
4	Content Words	duration	0.070	0.002	0.000	***
4	Content Words	Speech Rate	0.023	0.011	0.037	*
4	Content Words	Pre‐Word Pause	−0.034	0.003	0.000	***
4	All Words	(Intercept)	0.098	0.051	0.071	
4	All Words	surprisal	0.019	0.006	0.002	**
4	All Words	Word Frequency	0.036	0.005	0.000	***
4	All Words	Previous Mention	−0.013	0.006	0.040	*
4	All Words	age	−0.002	0.012	0.850	
4	All Words	Sex=Male	−0.015	0.024	0.537	
4	All Words	Sex=Other	−0.046	0.085	0.593	
4	All Words	Max Intensity Baseline	0.242	0.004	0.000	***
4	All Words	duration	0.109	0.002	0.000	***
4	All Words	Speech Rate	0.012	0.012	0.316	
4	All Words	Pre‐Word Pause	−0.035	0.003	0.000	***

*Note*. ***: *p* < 0.001, **: *p* < 0.01, *: *p* < 0.05.

**Table A2:** Regression results for predicting Pitch Range

Context Len.	Subset	Predictor	Estimate	Std. Error	Pr(>|t|)	Sig.
0	Content Words	(Intercept)	0.505	0.191	0.008	**
0	Content Words	surprisal	0.037	0.005	0.000	***
0	Content Words	Word Frequency	−1.227	0.111	0.000	***
0	Content Words	Previous Mention	0.007	0.008	0.328	
0	Content Words	age	0.005	0.021	0.801	
0	Content Words	Sex=Male	0.093	0.044	0.038	*
0	Content Words	Sex=Other	−0.222	0.156	0.158	
0	Content Words	Pitch Range Baseline	−2.540	0.017	0.000	***
0	Content Words	duration	0.142	0.004	0.000	***
0	Content Words	Speech Rate	0.007	0.021	0.740	
0	Content Words	Pre‐Word Pause	0.003	0.004	0.451	
0	All Words	(Intercept)	0.293	0.165	0.076	
0	All Words	surprisal	0.045	0.005	0.000	***
0	All Words	Word Frequency	−1.343	0.098	0.000	***
0	All Words	Previous Mention	0.015	0.007	0.021	*
0	All Words	age	0.008	0.019	0.668	
0	All Words	Sex=Male	0.084	0.039	0.036	*
0	All Words	Sex=Other	−0.052	0.139	0.712	
0	All Words	Pitch Range Baseline	−2.609	0.017	0.000	***
0	All Words	duration	0.216	0.003	0.000	***
0	All Words	Speech Rate	−0.003	0.019	0.869	
0	All Words	Pre‐Word Pause	0.007	0.003	0.039	*
4	Content Words	(Intercept)	0.515	0.191	0.007	**
4	Content Words	surprisal	0.032	0.005	0.000	***
4	Content Words	Word Frequency	−1.230	0.112	0.000	***
4	Content Words	Previous Mention	0.010	0.008	0.169	
4	Content Words	age	0.003	0.021	0.871	
4	Content Words	Sex=Male	0.087	0.043	0.047	*
4	Content Words	Sex=Other	−0.199	0.153	0.197	
4	Content Words	Pitch Range Baseline	−2.544	0.017	0.000	***
4	Content Words	duration	0.142	0.004	0.000	***
4	Content Words	Speech Rate	0.010	0.021	0.635	
4	Content Words	Pre‐Word Pause	0.002	0.004	0.500	
4	All Words	(Intercept)	0.294	0.166	0.076	
4	All Words	surprisal	0.041	0.004	0.000	***
4	All Words	Word Frequency	−1.349	0.098	0.000	***
4	All Words	Previous Mention	0.019	0.007	0.005	**
4	All Words	age	0.009	0.019	0.640	
4	All Words	Sex=Male	0.086	0.040	0.033	*
4	All Words	Sex=Other	−0.093	0.140	0.508	
4	All Words	Pitch Range Baseline	−2.612	0.017	0.000	***
4	All Words	duration	0.217	0.003	0.000	***
4	All Words	Speech Rate	−0.004	0.019	0.854	
4	All Words	Pre‐Word Pause	0.007	0.003	0.048	*

*Note*. ***: *p* < 0.001, **: *p* < 0.01, *: *p* < 0.05.

**Table A3:** Regression results for predicting Max Pitch

Context Len.	Subset	Predictor	Estimate	Std. Error	Pr(>|t|)	Sig.
0	Content Words	(Intercept)	0.781	0.168	0.000	***
0	Content Words	surprisal	0.032	0.006	0.000	***
0	Content Words	Word Frequency	−0.396	0.096	0.000	***
0	Content Words	Previous Mention	0.000	0.007	0.993	
0	Content Words	age	−0.004	0.021	0.837	
0	Content Words	Sex=Male	−0.454	0.044	0.000	***
0	Content Words	Sex=Other	−0.500	0.157	0.002	**
0	Content Words	Max Pitch Baseline	−1.912	0.014	0.000	***
0	Content Words	duration	0.059	0.003	0.000	***
0	Content Words	Speech Rate	0.023	0.021	0.277	
0	Content Words	Pre‐Word Pause	0.015	0.003	0.000	***
0	All Words	(Intercept)	0.599	0.145	0.000	***
0	All Words	surprisal	0.041	0.007	0.000	***
0	All Words	Word Frequency	−0.508	0.084	0.000	***
0	All Words	Previous Mention	0.003	0.006	0.649	
0	All Words	age	−0.006	0.023	0.787	
0	All Words	Sex=Male	−0.525	0.047	0.000	***
0	All Words	Sex=Other	−0.453	0.165	0.007	**
0	All Words	Max Pitch Baseline	−1.938	0.015	0.000	***
0	All Words	duration	0.106	0.003	0.000	***
0	All Words	Speech Rate	0.030	0.022	0.186	
0	All Words	Pre‐Word Pause	0.032	0.003	0.000	***
4	Content Words	(Intercept)	0.726	0.168	0.000	***
4	Content Words	surprisal	0.031	0.007	0.000	***
4	Content Words	Word Frequency	−0.408	0.097	0.000	***
4	Content Words	Previous Mention	0.003	0.007	0.626	
4	Content Words	age	0.000	0.019	0.992	
4	Content Words	Sex=Male	−0.362	0.040	0.000	***
4	Content Words	Sex=Other	−0.404	0.146	0.007	**
4	Content Words	Max Pitch Baseline	−1.921	0.014	0.000	***
4	Content Words	duration	0.059	0.003	0.000	***
4	Content Words	Speech Rate	0.020	0.019	0.308	
4	Content Words	Pre‐Word Pause	0.015	0.003	0.000	***
4	All Words	(Intercept)	0.549	0.145	0.000	***
4	All Words	surprisal	0.041	0.007	0.000	***
4	All Words	Word Frequency	−0.523	0.084	0.000	***
4	All Words	Previous Mention	0.007	0.006	0.266	
4	All Words	age	−0.005	0.022	0.830	
4	All Words	Sex=Male	−0.456	0.045	0.000	***
4	All Words	Sex=Other	−0.380	0.159	0.019	*
4	All Words	Max Pitch Baseline	−1.944	0.015	0.000	***
4	All Words	duration	0.106	0.003	0.000	***
4	All Words	Speech Rate	0.027	0.021	0.205	
4	All Words	Pre‐Word Pause	0.032	0.003	0.000	***

*Note*. ***: *p* < 0.001, **: *p* < 0.01, *: *p* < 0.05.

**Table A4:** Regression results for predicting Duration

Context Len.	Subset	Predictor	Estimate	Std. Error	Pr(>|t|)	Sig.
0	Content Words	(Intercept)	1.462	0.296	0.000	***
0	Content Words	surprisal	0.023	0.006	0.000	***
0	Content Words	Word Frequency	−4.640	0.177	0.000	***
0	Content Words	Syllable Count	0.043	0.012	0.000	***
0	Content Words	Previous Mention	−0.024	0.007	0.001	**
0	Content Words	age	−0.008	0.005	0.100	
0	Content Words	Sex=Male	−0.024	0.010	0.019	*
0	Content Words	Sex=Other	−0.045	0.038	0.238	
0	Content Words	Duration Baseline	−5.554	0.028	0.000	***
0	Content Words	Speech Rate	−0.091	0.005	0.000	***
0	Content Words	Pre‐Word Pause	−0.007	0.003	0.025	*
0	All Words	(Intercept)	1.201	0.253	0.000	***
0	All Words	surprisal	0.025	0.005	0.000	***
0	All Words	Word Frequency	−4.873	0.154	0.000	***
0	All Words	Syllable Count	0.040	0.010	0.000	***
0	All Words	Previous Mention	−0.027	0.006	0.000	***
0	All Words	age	−0.000	0.003	0.890	
0	All Words	Sex=Male	0.001	0.006	0.840	
0	All Words	Sex=Other	−0.021	0.022	0.352	
0	All Words	Duration Baseline	−5.688	0.025	0.000	***
0	All Words	Speech Rate	−0.090	0.003	0.000	***
0	All Words	Pre‐Word Pause	0.004	0.003	0.147	
4	Content Words	(Intercept)	1.418	0.297	0.000	***
4	Content Words	surprisal	0.021	0.005	0.000	***
4	Content Words	Word Frequency	−4.674	0.178	0.000	***
4	Content Words	Syllable Count	0.043	0.011	0.000	***
4	Content Words	Previous Mention	−0.021	0.007	0.004	**
4	Content Words	age	−0.007	0.005	0.133	
4	Content Words	Sex=Male	−0.022	0.010	0.027	*
4	Content Words	Sex=Other	−0.043	0.038	0.256	
4	Content Words	Duration Baseline	−5.556	0.028	0.000	***
4	Content Words	Speech Rate	−0.089	0.005	0.000	***
4	Content Words	Pre‐Word Pause	−0.007	0.003	0.021	*
4	All Words	(Intercept)	1.172	0.254	0.000	***
4	All Words	surprisal	0.023	0.004	0.000	***
4	All Words	Word Frequency	−4.899	0.155	0.000	***
4	All Words	Syllable Count	0.039	0.010	0.000	***
4	All Words	Previous Mention	−0.024	0.006	0.000	***
4	All Words	age	0.000	0.003	0.959	
4	All Words	Sex=Male	0.002	0.006	0.692	
4	All Words	Sex=Other	−0.020	0.022	0.381	
4	All Words	Duration Baseline	−5.690	0.025	0.000	***
4	All Words	Speech Rate	−0.090	0.003	0.000	***
4	All Words	Pre‐Word Pause	0.004	0.003	0.156	

*Note*. ***: *p* < 0.001, **: *p* < 0.01, *: *p* < 0.05.

## Appendix B Impact of “previous mention” on model fit

Fig. B1 compares the Akaike Information Criterion (AIC) across different context lengths, with and without the “previous mention” variable.

## Appendix C Comparing CANDOR default timestamps with Montreal Forced Aligner

Fig. C1 compares the distribution of word durations under the default timestamps from CANDOR (using AWS Transcribe) and Montreal Forced Aligner.

## Appendix D Evaluation of automated transcriptions

To evaluate the quality of the transcriptions provided via AWS Transcribe as part of CANDOR, we performed a manual transcription on a small subset of the data (two randomly selected 3‐min segments of conversation from two different conversations). Annotators were given the following instructions:

- Put all annotations in a blank text file, with one turn per line (i.e., move to a new line when the speaker changes—but see note below about backchannels).
- Please do NOT include listener backchannels in the transcript (i.e., when a listener says something like “yeah,” “mhm” without taking the floor from the speaker, just ignore this).
- Otherwise, transcribe what you hear, including filler words (“um,” “like”) and false starts.
- If you are unsure of what word was said, just put your best guess (do not put meta‐level annotations such as “unintelligible”).
- For evaluating word error rate, we will normalize the transcripts for case and remove punctuation (other than word‐internal punctuation like apostrophes within contractions), so there is no need to worry about these.

We compare the similarity of two transcriptions using Word Error Rate (WER), after normalizing the transcripts by removing capitalization and punctuation. WER is not symmetric, so for a given pair of transcriptions A and B, we take the mean of the WER when A is the reference and B is the hypothesis, and vice versa, to form a symmetric similarity metric. Our results, visualized as a distance matrix in Fig. D1, show that the ASR transcriptions have comparable mean WER relative to the interhuman average. Specifically, the mean WER for Human 1, Human 2, Human 3, Human 4, and ASR, respectively, were 0.15332912, 0.23281467, 0.18245587, 0.16327848, and 0.19404922. Thus, the mean WER of the ASR transcript to the other transcripts was less than the highest mean WER of each of the human transcripts to other transcripts. We also convert these pairwise distances into a two‐dimensional space using multi‐dimensional scaling (MDS), which we plot in Fig. D2. The MDS visualization shows a cluster of three human annotators who are closer to each other than to the ASR system, but another human who is even farther away. We note that this analysis is limited by a small number of annotators and a small sample of conversation snippets considered, but the results provide a basic sanity‐check for the quality of the ASR transcripts.
